# The combined effects of neurostimulation and priming on creative thinking. A preliminary tDCS study on dorsolateral prefrontal cortex

**DOI:** 10.3389/fnhum.2015.00403

**Published:** 2015-07-17

**Authors:** Barbara Colombo, Noemi Bartesaghi, Luisa Simonelli, Alessandro Antonietti

**Affiliations:** ^1^Department of Psychology, Catholic University of the Sacred HeartMilano, Italy; ^2^Division of Education and Human Studies, Champlain CollegeBurlington, VT, USA

**Keywords:** neurostimulation, tDCS, creativity, divergent thinking, attention, prefrontal cortex, biofeedback, skin temperature

## Abstract

The role of prefrontal cortex (PFC) in influencing creative thinking has been investigated by many researchers who, while succeeding in proving an effective involvement of PFC, reported suggestive but sometimes conflicting results. In order to better understand the relationships between creative thinking and brain activation in a more specific area of the PFC, we explored the role of dorsolateral PFC (DLPFC). We devised an experimental protocol using transcranial direct-current stimulation (tDCS). The study was based on a 3 (kind of stimulation: anodal vs. cathodal vs. sham) × 2 (priming: divergent vs. convergent) design. Forty-five healthy adults were randomly assigned to one stimulation condition. Participants’ creativity skills were assessed using the Product Improvement subtest from the Torrance Tests of Creative Thinking (TTCT). After 20 min of tDCS stimulation, participants were presented with visual images of common objects. Half of the participants were instructed to visualize themselves using the object in an unusual way (divergent priming), whereas the other half were asked to visualize themselves while using the object in a common way (convergent priming). Priming was aimed at inducing participants to adopt different attitudes toward the creative task. Afterwards, participants were asked to describe all of the possible uses of the objects that were presented. Participants’ physiological activation was recorded using a biofeedback equipment. Results showed a significant effect of anodal stimulation that enhanced creative performance, but only after divergent priming. Participants showed lower skin temperature values after cathodal stimulation, a finding which is coherent with studies reporting that, when a task is not creative or creative thinking is not prompted, people show lower levels of arousal. Differences in individual levels of creativity as assessed by the Product Improvement test were not influential. The involvement of DLPFC in creativity has been supported, presumably in association to shift of attention modulated by priming.

## Introduction

Many studies about the neurobiological counterparts of creativity have been recently published (for reviews, see Heilman, 2005; Skov and Vartanian, 2009; Dietrich and Kanso, 2010; Kaufman et al., 2010; Sawyer, 2011; Abraham, 2013; Jung et al., 2013). A notable number of them focused on exploring the contributions of the prefrontal cortex (PFC) in modulating creativity (Solso, 2001; Brown et al., 2006; Bengtsson et al., 2007; Berkowitz and Ansari, 2008; Kowatari et al., 2009), supporting the notion that such a brain area is involved in creative thinking (Fink et al., 2009a; Shamay-Tsoory et al., 2011; Green et al., 2012). It is interesting to note that studies generally failed to reveal differential activation across the two hemispheres: the left and right PFC appear to be equally activated in most creative tasks (Bekhtereva et al., 2000, 2001; Carlsson et al., 2000; Sawyer, 2006, 2011; Runco, 2007; de Souza et al., 2010; Benedek et al., 2011). These findings are consistent with studies investigating other brain areas (Badzakova-Trajkov et al., 2011) and with a meta-analytic review of the literature, which failed to support the notion that creativity is associated to a specific cerebral hemisphere (Mihov et al., 2010). Heilman et al. (2003) found that inter-hemispheric communication is important especially in promoting creative innovation, thus supporting the notion that creativity is associated with the integration of conceptually distant ideas held in different brain domains, as suggested by Takeuchi et al. (2010).

The involvement of PFC in creativity was also supported by clinical studies. As reported in a recent review (Szczepanski and Knight, 2014), there is evidence that damage in rostral PFC causes impairments in creativity and the amount of hypoperfusion in PFC predicts scores in the Torrance Tests of Creative Thinking (TTCT; Torrance, 1990). Shamay-Tsoory et al. (2011) found that greater lesion volume in medial BA10 predicted greater impairments in creativity and original thinking, as measured by TTCT. Patients with the frontal variant of frontotemporal lobar degeneration, which causes bilateral degeneration of anterior prefrontal and temporal cortex often disproportionately affecting the ventromedial PFC, are also severely impaired on TTCT (de Souza et al., 2010).

PFC, however, is a part of the cerebral cortex that serves many cognitive functions. Therefore, identifying more detailed brain regions supporting the generation of novel and original ideas has been stressed as a need for future investigations (Arden et al., 2010). A putative candidate that might help to better understand the specific role that brain structures and networks play in creative thinking is dorsolateral PFC (DLPFC; Dietrich, 2004). Right DLPFC has been showed to be implied in ill-structured problems that require finding unusual solutions (Gilbert et al., 2010). Left DLPFC was found to be activated by a divergent task (i.e., a task that requires thinking in an unusual way, for example to generate several possible solutions to a problem; Vartanian and Goel, 2007). Moreover, goal-directed planning of novel solutions during a creative task was reported to be organized top-down thanks to the contribution of left DLPFC (Aziz-Zadeh et al., 2013). The role of DLPFC in creative thinking depends presumably on the combined result of several of its functions, which include the ability to focus or defocus attention and to shift the focus of attention (Prakash and Du, 2013).

Dietrich and Kanso (2010) stressed some methodological issues linked to the study of the neurobiological bases of creativity. These caveats should be taken into account while planning a study addressing this topic. The authors claimed that, even if neuroelectric and imaging studies of the neural bases of creativity are consistent in reflecting changes in prefrontal areas, taking all the studies together, creative thinking associated with PFC does not appear to critically depend on any single mental process.

To make creativity tractable in this brain region, apart from focusing on a more specific area as discussed above, creativity should also be further subdivided into different types that can be meaningfully associated with specific cognitive processes. To achieve this goal, the use of tasks that allow investigators to decompose the creative process into specific factors maybe useful. Yet, most studies in which the association between DLPFC and creativity was investigated failed to face participants with tasks that are usually employed to assess creative skills. Thus, we aimed at investigating the role of DLPFC in the execution of a task which is often used both in testing creativity abilities in general (it is included in TTCT, the most widely employed measure of creative skills) and in investigating the neural correlates of creativity (e.g., Carlsson et al., 2000; Fink et al., 2007, 2009a,b; de Souza et al., 2010; Shamay-Tsoory et al., 2011), namely, the request to list as many uses as possible of a given object (alternate uses—AU—task). The reason why TTCT is so widely used is that it allows researchers to assess three main creativity factors (Guilford, 1967; Torrance, 1990): fluidity (also called fluency), flexibility and originality. Fluidity is defined as the ability to produce numerous ideas in relation to a given situation or problem. Flexibility represents the level of ability in changing thinking strategy. Originality refers to the ability in findings ideas that other people fail to generate.

### Neurostimulation Studies about Creativity

In our study, we decided to use neurostimulation to test a causal link, and not only correlations (as found through EEG, PET or fMRI), between DLPFC and creativity (Iannello et al., 2014). Transcranial direct current stimulation (tDCS) is one of the most common technique for non-invasive brain stimulation (together with transcranial magnetic stimulation, TMS). It is silent and painless and it modulates the spontaneous neuronal activity in a given brain region through the constant intensity of an electrical current, delivered via small electrodes, that produces a temporary hypo/hyperactivity in that region. The positive pole (anodal stimulation) increases the excitability of neural tissues, whereas the negative (cathodal stimulation) decreases it (Nitsche and Paulus, 2000; Nitsche et al., 2008; Galea et al., 2009). During tDCS, low-amplitude direct currents are applied via scalp electrodes and penetrate the skull to enter the brain. These currents modify the transmembrane neuronal potential and thus influence the level of excitability and modulate the firing rate of neurons in response to additional inputs (Wagner et al., 2007). Using non-invasive brain stimulation to examine the causal relationships between reduced/enhanced function of the PFC and performance in tasks involving different kinds of cognitive skills has been proven to be effective in previous studies (Leite et al., 2011, 2013; Coffman et al., 2014).

As creativity is concerned, Chrysikou et al. (2013) found a significant facilitative effect of left PFC cathodal stimulation for listing uncommon tool uses. They read this result as supporting the claim that certain tasks may benefit from a state of diminished cognitive control. Cerruti and Schlaug (2009) found that anodal tDCS over left DLPFC increased performance in the Remote Associates Test (RAT; Mednick and Mednick, 1967), a task requiring respondents to find a word which can be linked to three given words (for instance, given the words “crab”, “pine” and “sauce”, the to-be-found word is “apple” since it can be associated to those words because it is included in the words “crabapple”, “pineapple” and “applesauce”), as compared to cathodal and sham stimulation. However, as claimed by the authors themselves, RAT is a complex task involving not only creative skills, but also general intelligence (and verbal competence: Mendelsohn, 1976), so that it is sometimes meant as a measure of analytical and deductive, rather than creative, thinking (Zmigrod et al., in press). In addition, Cerruti and Schlaug (2009) findings failed to be replicated by Metuki et al. (2012). A genuine creativity test, namely, the AU task, was employed by Zmigrod et al. (in press) in a within-subject experiment. They reported that the combination of anodal stimulation of left DLPFC and cathodal stimulation of right DLPFC, which produced a better performance in a revised version of RAT, increased fluidity, flexibility and elaboration scores in the AU task, as well in comparison to the opposite montage and to the sham condition, even though differences were not statistically significant.

### Priming as a Procedure to Investigate Creativity

Recently the effects of priming, intended as a way to induce individuals to adopt a particular attitude toward a task on creativity have been addressed as an implicit way to enhance performance (e.g., Förster et al., 2009; Baas et al., 2011; Litchfield et al., 2011; Bittner and Heidemeier, 2013; González-Gómez and Richter, 2015). Since the early sixties Mednick et al. (1964) reported an effect of specific associative priming upon incubation of creative performance and Torrance (1961) discussed the positive effects of different types of priming to improve primary school children’s creativity. More recently, Rietzschel et al. (2007) found that using priming (referring to specific sub-categories of a brainstorming task) before a creative activity caused a higher productivity and originality in participants’ responses. Among the different procedures to prime people, mental simulation appears to be a promising one. For example, Elder and Krishna (2012) showed that asking participants to visually depict themselves while using an artifact in different ways results in different behaviors. Even though their study was not focused on creativity *per se*, if paired with the results from the other studies mentioned above, it suggests an interesting methodological approach. Priming appears to be useful to cause different attitudes, and consequently behaviors, toward a task and hence it could be relevant to explore in a more focused way the effect of neurostimulation.

The studies mentioned above used a promising design but still present a few weaknesses. Elder and Krishna (2012) used food-related visual stimuli. Participants’ answers to these stimuli may be influenced by personal preferences, habits (which can modify the perceived hunger), experiment timing (e.g., participants may be more activated in response to food-related stimuli close to lunch time; Killgore et al., 2013) or type of food (more or less caloric; Lietti et al., 2012). Since the authors did not control for these variables, their results may not be easily generalized. Chrysikou et al. (2013) overcame some of these limitations using more ecological stimuli (everyday objects). Yet, their stimuli were presented in gray-scale, thus reducing ecological validity. This is a fact which cannot be ignored, since Amsel et al. (2014), by recording event-related potentials while manipulating visual contrast, found that accessing information about colors plays a role in accessing object knowledge.

### Physiological Activation and Creativity

Literature suggests that there may be differences in physiological activation in relation to individual creativity levels or to the task characteristics: more creative individuals, or individuals performing better at a creative task, show lower body temperature (De Dreu et al., 2008, 2010; Colombo et al., 2013). Environmental and mood condition that are linked to lower physiological activation have also been proved by a meta-analysis to be linked to worse creative performance (Baas et al., 2008). Bazanova and colleagues further supported the link between brain activation, physiological activation and creative performance. Prior research showed that using biofeedback to modulate physiological activation affects the alpha-activity and, ultimately, the creative performance in a specific population (musicians; Bazanova et al., 2007, 2009). Similar results were also found when working with different target populations. For example, Oded (2011) reported a similar connection between physiological activation (as recorded by biofeedback) and cognitive flexibility in military personnel. This finding, which has not yet been widely explored in literature, is particularly interesting because the reasons for different activation patterns in more creative individuals can be associated with different activation patterns of the PFC. The role of the PFC in modulating physiological responses has been stressed by Nagai et al. (2004), who observed that activity in ventromedial PFC covaries with skin conductance level. Starting from these data, we decided to further explore the influence of tDCS on physiological activation, using biofeedback techniques to record participants’ skin temperature variations while they were performing the creative task.

## Aims and Hypotheses

In the light of the existing literature, the goal of the present study was to deepen the understanding of the role of DLPFC in creative performance by means of tDCS.

Similarly to Chrysikou et al. (2013) and to Zmigrod et al. (in press), we employed the AU task, which is commonly recognized as a relevant task to highlight to what extent people are able to generate novel and useful ideas, but not the RAT (as Cerruti and Schlaug, 2009, did), which is a mixed measure of different skills. Whereas Chrysikou et al. (2013) measured only reaction times in the AU task, we, analogously to what Zmigrod et al. (in press) did, analyzed the quality of the responses by assessing fluidity, flexibility and originality [which was not scored by Zmigrod et al. (in press), who instead scored elaboration, which however is not so crucial for creativity].

As Cerruti and Schlaug (2009) and Zmigrod et al. (in press), but not Chrysikou et al. (2013; who applied only cathodal stimulation), did, we tested the effects of both stimulation inhibiting (cathodal) and enhancing (anodal) brain activity with reference to a control (sham) condition. We choose to apply only a kind of stimulation (either anodal or cathodal), as Cerruti and Schlaug (2009) and Chrysikou et al. (2013) did, but not the concurrent, inverse stimulation of bilateral brain areas as Zmigrod et al. (in press) did, in order to clarify the isolated role of each kind of stimulation, by avoiding interfering effects due to the simultaneous alteration of brain functioning produced in the opposite hemisphere, and to make results comparable to the previous ones acquired through the same procedure in more than one study. For the same reason we choose to implement a between-subject experimental design, as Cerruti and Schlaug (2009) and Chrysikou et al. (2013) did, but not a within-subject design as Zmigrod et al. (in press) did, which might produce learning effects due to the repeated exposure to the same kind of task.

We introduced a new manipulation in research of creativity through neurostimulation, that is, priming, which has been showed to be a promising procedure to investigate the creative process and its enhancement. We made reference to the procedure devised by Elder and Krishna (2012), but changed the materials by controlling the features of the stimuli so to improve external validity.

Finally, we recorded also participants’ creativity skills and physiological measures so to assess, respectively, the role of the individual level of creativity, that was treated as a covariate in our analyses, in order to be able to rule out possible disturbing effects of individual differences (something that previous studies did not control for) and the modulation of activation across the creative task. These aspects have been never investigated before in association to neurostimulation.

Our hypotheses were as follows:

1. *Effect of tDCS on creative performance*. Anodal stimulation of DLPFC should promote creative performance since, according to literature (Cerruti and Schlaug, 2009; Zmigrod et al., in press), this specific area tends to be positively associated with a better creative performance.
2. *Effect of the priming on creative performance*. Divergent mental simulation was expected to promote more creative answers in the AU task. We expected participants to report more (increased fluidity) and more original (increased originality) uses. We also expected them to be more flexible, changing their strategy of thinking more often.
3. *Effect of tDCS on physiological activation during the creative task*. Literature, even though with some inconsistencies, suggests an inverse relationship between creativity and activation. Thus, we expected to record higher activation after convergent priming and cathodal stimulation, which, by inhibiting DLPFC, should decrease the level of creativity, and lower activation after divergent priming and anodal stimulation, which should enhance creative performance thanks to DLPFC hyperactivation.

## Methods

### Tools

#### Assessment of Individual Creativity Skills

To assess the individual level of creativity, we used the Product Improvement test, one of the verbal tasks based on non-verbal stimuli that are included in TTCT. In this task, a picture of a common toy (stuffed animal) is shown and respondents are asked to think of as many improvements as they can which would make the toy “more fun to play with”. Researchers coded each answer, assigning three scores related to:

a. *Fluidity*: the ability to produce numerous ideas in relation to a given situation or a problem. This factor corresponds to the number of relevant answers provided by the participant.
b. *Flexibility*: the ability to change thinking strategy, moving from one conceptual category to another. Flexibility is computed by counting the number of categories within which each response can be classified. Researchers assigned a point for each answer that belongs to a different category.
c. *Originality*: the ability to find unusual answers, which few others would think of. For each answer, researchers assigned a score from 0 to 3. The lowest score corresponds to usual and common ideas whereas the highest score is assigned to uncommon and original answers. The total Originality score is obtained by adding up the Originality scores of each response. To assign originality scores, we used the reference tables provided with the test. We checked the distribution of scores in our sample and it did not differ significantly from the one of the standardized Italian version of the test.

A total creativity score, derived by adding up the weighted scores of the three factors mentioned above, was also computed. We had to weight the scores, since the ranges for the different scores varied and we did not want the total score to be biased by the width of the range of scores of the distinct factors.

Two independent judges evaluated participants’ performance. Inter-rater reliability was 0.79. We used the mean scores of their evaluations for statistical analyses.

#### Neurostimulation

The tDCS equipment used in the study (HDC Series by Newronika S.r.l, Milano) is composed of two sponge-based electrodes (25 cm^2^): one (either the anodal or the cathodal one, according to the specific stimulation condition) is positioned on the subject’s scalp and the other on the ipsilateral mastoid, in order to constrain tDCS application to one hemisphere. This specific montage has been used in previous studies (e.g., Beeli et al., 2008; Zaehle et al., 2011; Asthana et al., 2013) and its efficacy has been discussed in a recent review (Tremblay et al., 2014). Participants’ DLPFC, identified through the 10–20 EEG international system (F4 electrode position for the RPFC and F3 electrode position for LPFC), was stimulated at a constant current of 1.5 mA for 20 min. Previous experiments have shown that this stimulation duration induces cortical excitability shifts that are stable for at least 1 h after the end of neurostimulation (Nitsche and Paulus, 2000; Nitsche et al., 2003). In the anodal condition, the anode electrode was positioned on F4 or F3 and the cathode electrode on the ipsilateral mastoid. In the cathodal condition, the two electrodes were switched (cathode over F4 or F3, anode over ipsilateral mastoid). For each condition half participants (seven or eight) received stimulation over F4 and the other half (seven or eight) over F3.

#### Biofeedback

Physiological measures were recorded using a biofeedback equipment (model 2000^x-pert^ by Schufried GmbH, Austria). This is a non-invasive instrument that monitors and records an individual’s physiological activity. Thanks to a sensor connected to the participant’s finger, physiological indices—in our case skin temperature (TEMP)—are recorded and directly delivered to the 2000^x-pert^ software via Bluetooth and visually displayed on a computer monitor supervised by the experimenter for the duration of the entire session. Participants did not have any access to this information any time during the experiment. The sensor does not cause any pain or discomfort. Temperature is recorded by this equipment in Celsius degree, four values per second, with a range between 10 and 40°C and a resolution of 0.01°C.

#### Priming and Creative Tasks

Nine images of commonly used artifacts to use as stimuli for the creative task were selected (see “Procedure” Section for details about how they were used). All images were actual advertisements of common objects, in order to increase the ecological validity of the study and they all were placed against a similar neutral background in order to allow comparison among the different objects. The objects used were a mug, some markers, sunglasses, a razor, a keychain, a watch, a portable umbrella, a coffee maker, a sleeping mask. Unlike Elder and Krishna (2012), we did not select food-related images because responses to those may be biased, as detailed in the “Introduction” Section. We also selected products that were not likely to induce any gender-biased responses.

We used a mental simulation task as priming. The main aim of the priming was to induce participants to adopt different attitudes toward the creative task (listing as many uses as possible of the displayed object): either to “converge” on the common use or to “diverge” by thinking of an uncommon use. Information about the priming was given to each participant before the creative task was begun. In the divergent priming condition, participants were asked to visualize themselves while using the presented object in an unusual way. In the convergent condition, participants were asked to visualize themselves while using the object in the traditional way. In both cases, participants were instructed to form in their mind a clear visual image of themselves while using the object and to keep it until the subsequent request. Ten seconds for each object were devoted to mental simulation in both conditions. The timing started immediately after each object was shown; after the 10 s the AU task began and participants were asked by the researchers to list all the possible uses of the object they saw before (see “Procedure” Section for more details). To be sure that participants actually followed the instructions and visualized just one use for each object, they were asked to verbalize what they were imagining.

### Sample

Forty-five participants (41 women and 4 men, between the ages of 18 and 27 years, *M* = 22.86, SD = 1.85) volunteered to participate in the study. They were not paid and did not receive any course credit for their participation.

They were randomly assigned to one of three stimulation conditions: anodal (A-tDCS), cathodal (C-tDCS) or control (sham) stimulation. We had 15 participants for each stimulation condition, a number that fits the requirements for this kind of study [for instance, in Chrysikou et al. (2013) study, eight participants were allocated to each stimulation condition].

We found no significant difference (M_Anodal_ = 19.31; SD_Anodal_ = 10.01; M_Cathodal_ = 16.38; SD_Cathodal_ = 9.35; M_Sham_ = 13.40; SD_Sham_ = 5.60; *F*_(2,41)_ = 1.06, *p* = 0.36) among the three samples according to their scores in the creativity levels as measured through the Product Improvement test, so we assumed that the three groups were homogenous with respect to their individual creativity levels.

Participants were screened before the experiment to exclude any possible neurological, cognitive or visual deficit.

The study has been revised and approved by the ethical committee of the institution where the experiment took place.

### Procedure

Participants’ creativity levels were assessed using the Product Improvement test 5 days before the actual experiment, in order to avoid any interference or familiarity effect on the performance in the AU task.

On the day of the experiment, each participant was given the informed consent form to read and sign. Researchers answered any questions about the experimental procedure at the time the form was signed.

After signing the informed consent form, participants’ biofeedback baselines were recorded. Participants’ physiological indexes were recorded for 2 min. In order to record signals without any possible external interference, individuals were asked to relax and look at landscape images that were presented on a computer screen.

After this preliminary phase, participants went through 20 min of tDCS stimulation (either anodal, cathodal or sham). During the stimulation, they were engaged in a relaxing task (reading a travel diary) unrelated to the priming and AU tasks. Unlike methods used in other studies, we decided to stimulate the DLPFC before, but not during the task. We know, as detailed above, that tDCS effects on the PFC can last for at least 1 h after the end of tDCS (e.g., Nitsche and Paulus, 2000; Nitsche et al., 2003) and decided that participants would likely feel more comfortable performing a creative task without the electrodes.

Following the stimulation, participants were presented with visual images of objects. The images were presented to participants in a random order. Each image was shown for 10 s. For the priming task, half of the participants were instructed to visualize themselves (for 10 s) using the object in an unusual way (divergent priming), whereas the other half were asked to visualize themselves using the object in a common way (convergent priming). They had to describe out aloud the visualized image. Afterwards participants were asked to describe out aloud all the possible uses of the object they just saw for 10 s (AU task). The same procedure was repeated for each of the nine objects. The experimenter overseeing the session recorded the participant’s answers.

Creativity scores related to the AU task were computed using the same procedure and criteria used for the Product Improvement test described above. To be more precise, researchers coded each answer assigning three scores related to:

a. *Fluidity*: the number of relevant answers provided by the participant.
b. *Flexibility*: the number of categories within which each response can be classified. Researchers assigned a point for each answer that belongs to a different category. A total score was computed by summing up scores for all the nine objects.
c. *Originality*: for each answer, researchers assigned a score from 0 to 3. The lowest score corresponds to usual and common ideas while the highest score is assigned to uncommon and original answers. The total Originality score was obtained by adding up the Originality scores of each response to that item. A total score was computed for all the nine objects.
d. *Total creativity score*, derived by adding up weighted scores of the three factors mentioned above, was also computed. As mentioned above, we had to weight the scores, since the ranges for each score varied.

Two independent judges evaluated participants’ performance. Inter-rater reliability was 0.75. We used the mean scores of their evaluations for statistical analyses.

Participants’ physiological indexes were recorded during the entire task.

**Figure 1:**
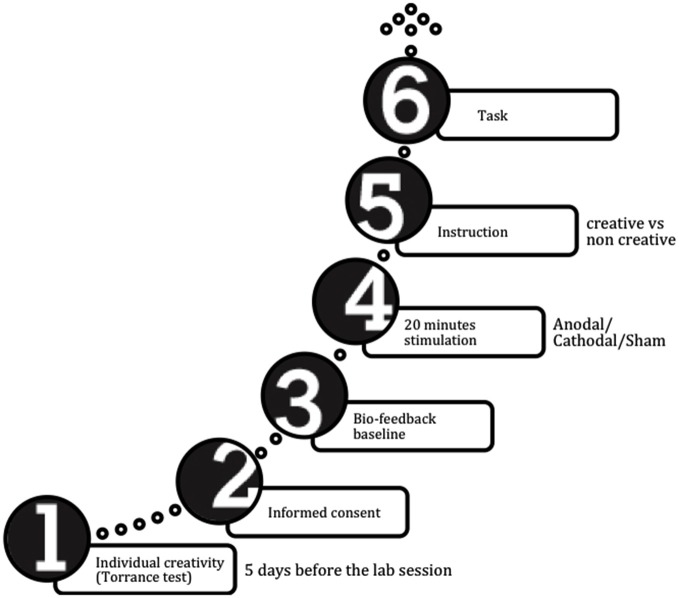
**Procedure timeline**.

A schematic timeline of the procedure is reported in Figure 1.

## Results

As a first step we focused on the priming task by looking for evidence that participants performed it following the instructions and it did not interfere with the AU task. A first evidence could be derived by the fact that creative answers after divergent priming tended to reorganize the stimulus to a wider extent (e.g., a creative use of razor after divergent simulation would be “a slide for ants”, a creative use of razor after convergent simulation would be “a spoon”). Non-creative answers tended to be similar after both priming (e.g., many participants suggested to use sunglasses as a hairband regardless of the priming condition).

Moreover, focusing on the results of the sham condition (where the effect of the priming *per se* could be tested), no significant difference emerged between the two priming conditions; actually participants performed slightly better after the convergent priming (see Tables 1, 2).

**Table 1:** **Total creativity scores (mean scores and standard deviations) according to the type of neurostimulation and priming**.

tDCS	Priming	Mean	Standard deviation
Anodal	Convergent	52.19	10.27
	Divergent	90.60	36.38
Cathodal	Convergent	55.08	22.40
	Divergent	62.65	21.77
Sham	Convergent	57.28	19.31
	Divergent	45.08	14.64

**Table 2:** **Creativity scores for single factors (mean scores and standard deviations) according to the type of neurostimulation and priming**.

	tDCS	Priming	Mean	Standard deviation
Fluidity	Anodal	Convergent	39.88	9.40
		Divergent	51.38	14.45
	Cathodal	Convergent	38.43	19.28
		Divergent	39.00	6.86
	Sham	Convergent	38.29	4.11
		Divergent	31.00	9.61
Flexibility	Anodal	Convergent	14.39	0.51
		Divergent	25.95	0.89
	Cathodal	Convergent	16.47	1.19
		Divergent	19.39	0.77
	Sham	Convergent	15.67	0.69
		Divergent	12.19	0.64
Originality	Anodal	Convergent	10.88	4.55
		Divergent	36.63	15.41
	Cathodal	Convergent	15.00	5.17
		Divergent	21.71	7.76
	Sham	Convergent	17.43	9.40
		Divergent	12.86	6.98

Preliminary analysis highlighted no significant difference between right and left stimulation of DLPFC. The effect of stimulation site on total creativity scores was not significant: *F*_(1,42)_ = 0.02; *p* = 0.88; η^2^ = 0.001 (M_left_ = 60.43, SD_left_ = 27.21; M_right_ = 61.61, SD_right_ = 28.45). The effect of stimulation site on physiological activation was not significant: *F*_(1,42)_ = 3.10; *p* = 0.08; η^2^ = 0.07 (M_left_ = −0.21, SD_left_ = 0.07; M_right_ = 0.25, SD_right_ = 0.09). Hence, we collapsed data and worked with a 3 (kind of neurostimulation: anodal vs. cathodal vs. sham) × 2 (priming: convergent vs. divergent) design.

### Effects of Neurostimulation and Priming on Creative Performance

We performed an ANCOVA on total creativity scores in the AU task (dependent variable) by considering type of stimulation and type of priming as independent variables and individual creativity levels (total score) as assessed through the Product Improvement test as a covariate. To avoid possible biases due to the sample size we performed a bootstrap, using 1000 bootstrap samples and 95% percentile confidence interval.^1^ Mean scores and SDs are reported in Table 1.

The between-subject test highlighted a main effect of the model (i.e., type of stimulation and type of priming were the independent variables that constituted the model) on the dependent variable (total creativity score; *F*_(6,37)_ = 3.60; *p* < 0.01; η^2^ = 0.37, *R*^2^ = 0.37) and a significant interaction effect of the stimulation type (anodal vs. cathodal vs. sham) × priming (divergent vs. convergent; *F*_(2,37)_ = 3.16; *p* < 0.05; η^2^ = 0.15). The Gabriel’s *post hoc* test (selected on the bases of the relatively small size of our sample) showed no significant difference between tDCS conditions (the main difference, even if it was not significant, was between anodal and sham condition: Mean difference = 20.21; SE = 8.94; *p* = 0.06). We also computed a series of *t*-tests aimed at assessing differences between the different levels of our two independent variables. The only significant difference that emerged was between anodal and sham conditions (*t*_28_ = 2.09, *p* < 0.05). No significant difference emerged between anodal and cathodal conditions (*t*_28_ = 1.06, *p* = 0.29), and between cathodal and sham conditions (*t*_28_ = 0.97, *p* = 0.34).

We believed that interaction effect that emerged from this first analysis needed to be clarified more focusing on the distinct creativity scores meant as a relevant way of assessing the individual contribution of each factor. Hence, we computed a MANCOVA using the distinct creativity scores (Fluidity, Flexibility and Originality) as dependent variables within the same general model and type of stimulation and type of priming as independent variables. Individual creativity levels, as assessed through the Product Improvement test, were used in the model as a covariate. To avoid possible bias due to the sample size we performed a bootstrap, using 1000 bootstrap samples and 95% percentile confidence interval. Mean scores and SDs are reported in Table 2.

The between-subject test highlighted a main effect of our independent variables (type of stimulation and type of priming) on all the dependent variables considered in the model: Fluidity (*F*_(6,37)_ = 3.52; *p* < 0.01; η^2^ = 0.36), Flexibility (*F*_(6,37)_ = 2.92; *p* < 0.05; η^2^ = 0.32) and Originality (*F*_(6,37)_ = 2.38; *p* < 0.05; η^2^ = 0.28). It is interesting to stress that the covariate (individual creativity level) had a significant effect only on Fluidity scores (*F*_(1,37)_ = 7.21; *p* < 0.05; η^2^ = 0.16). The Gabriel’s *post hoc* test showed a significant difference only between anodal and sham condition (Mean difference = 10.98; SE = 4.30; *p* < 0.05). As expected from the results previously reported, an interaction effect emerged in the between-subjects test: it is was related specifically to the Originality score (*F*_(2,37)_ = 2.77; *p* < 0.05; η^2^ = 0.19).

Considering the overall effects on the total creativity scores of the AU task, we can see a clear effect of anodal stimulation, but only after the divergent priming.

Focusing on the single factors, we observed that the general trend described before is always present after divergent priming, where anodal stimulation apparently improved performance.

### Effects of Neurostimulation and Priming on Physiological Activation

To exclude any possible influence due to the individual variability in skin temperature, as a preliminary step we run a regression on the temperature values recorded during the task, using the baseline values of the temperature for each participant as a predictor. We saved the standardized residuals and used these values to run our analyses.

We performed an ANCOVA on skin temperature values by considering the type of stimulation and the type of priming as independent variables. Individual levels of creativity were used as covariate. To avoid possible bias due to the sample size we performed a bootstrap, using 1000 bootstrap samples and 95% percentile confidence interval. Mean scores and SDs are reported in Table 3.

**Table 3:** **Body temperature values (mean scores and standard deviations of the standardized residuals derived by running a linear regression of the baseline temperature in Celsius degrees on the body temperature scores, in Celsius degrees, during the task) according to the type of neurostimulation and priming**.

tDCS	Priming	Mean	Standard deviation
Anodal	Convergent	0.39	0.06
	Divergent	0.15	0.08
Cathodal	Convergent	−0.19	0.06
	Divergent	−0.77	0.27
Sham	Convergent	0.28	0.09
	Divergent	−0.01	0.08

We found a significant effect of the stimulation (*F*_(2,37)_ = 3.27; *p* < 0.05; η^2^ = 0.18). Cathodal stimulation decreased temperature both after divergent and convergent priming (pairwise comparison: *t*_28_ = 2.54, *p* < 0.05), whereas the effects of anodal stimulation did not differ significantly from sham condition (pairwise comparison: *t*_28_ = 0.38 *p* = 0.71).

## Discussion and Conclusions

Neuroscience of creativity has become a relevant topic in the last years (Skov and Vartanian, 2009; Kaufman et al., 2010; Sawyer, 2011; Abraham, 2013). The role of PFC in influencing creative thinking has been explored by many researchers (Solso, 2001; Brown et al., 2006; Bengtsson et al., 2007; Berkowitz and Ansari, 2008; Kowatari et al., 2009) who, while succeeding in proving an effective involvement of PFC, failed to reveal a consistent differential activation across the two hemispheres: the left and right PFCs appear to be equally activated in most creative tasks (Carlsson et al., 2000; Bekhtereva et al., 2000, 2001; Sawyer, 2006, 2011; Runco, 2007; de Souza et al., 2010; Benedek et al., 2011).

In order to better explore the relationship between creative thinking and brain activation, we decided to focus on the specific role of DLPFC. We choose this area because its involvement in managing creative processes can be derived from previous studies (Vartanian and Goel, 2007; Gilbert et al., 2010; Aziz-Zadeh et al., 2013). Trying to derive a causal relationship between different patterns of brain activation and specific creative behaviors, we used neurostimulation. In order to devise an effective experimental design, we selected a task that relied on ecological stimuli and used a well-reputed and often employed tool that allowed us to work with specific and tractable constructs of creativity. We added, following the suggestions of previous studies, a priming before the actual creative task. The main aim of this priming was to induce participants to adopt different attitudes toward the creative task (listing as many uses as possible of the displayed object): either to “converge” on the common use or to “diverge” by thinking of an uncommon use. We also recorded body temperature during the AU task in order to collect data enabling us to enrich the corpus of information already reported in the literature. The role of individual levels of creativity has been taken into account as well.

Considering our hypotheses, the first and second ones appeared to be intertwined. The first hypothesis concerned the effect of tDCS on creative performance. Anodal stimulation did improve performance, but only after a divergent priming. We also conjectured that a priming task based on mental simulation would affect the AU task (second hypothesis). Participants did perform better after the divergent priming (the most affected factors were Originality and Flexibility), but this was true only for those who received anodal stimulation before the task. Apparently there is a relation between people’s attitude towards the task and the effect of neuromodulation. This finding is not only interesting but also relevant to better understand the actual effect of neuromodulation. As reported in a recent review (Ridding and Ziemann, 2010), even in neurologically normal subjects the variability in the neurophysiological and behavioral responses to brain stimulation techniques is high. The cause of this variability is multifactorial and to some degree still unknown. Our data could be read as a promising first step to identify one possible cause of this variability, namely, the individuals’ disposition towards the task.

The effects we found in relation to the first two hypotheses can be explained by referring to participants’ attentional focus, an issue which has been related to creativity (Ansburg and Hill, 2003). DLPFC is involved in attentional shift (Wager et al., 2004). Both Kondo et al. (2004) and Li et al. (2004) reported that DLFFC is a brain structure which is activated when individuals are asked to switch attention in working memory tasks. The same structure is involved in semantic memory (Nyberg et al., 2003). The role of DLPFC in attention shifting goes beyond memory and involves also central executive functions (Kane and Engle, 2002). Thus we can conjecture that the hyperactivation of DLPFC due to A-tDCS is beneficial in the AU task where respondents, in order to figure out original uses of objects, have to shift attention away from common uses and scan memory networks in search of less obvious ways of employing them. In other words, attentional shift might support the change of perspective (moving from thinking to common experience to imagining unusual possibilities) which is implied in the AU task, as well as in other creative tasks. According to the opportunistic-assimilation hypothesis (Seifert et al., 1995), a creative outcome emerges when the individual moves attention from the central elements of the situation to other aspects of the environment (Prakash and Du, 2013). It has been reported that people who lack the ability to keep attention focused and tend to move it away from the initial target are more creative (Necka, 1999; Carson et al., 2003). In addition, procedures aimed at priming participants to be center-focused has been showed to decrease originality in an AU task (Friedman et al., 2003) and to inhibit the solution of problems through insight (Wegbreit et al., 2012), a process which has been linked to creativity (Antonietti and Colombo, 2013). Being able to change the attentional focus can improve performance during the AU task because people can switch easily from the common use to the unusual ones.

The third hypothesis concerned the effect of tDCS on physiological activation during the creative task. We observed higher skin temperature in the convergent than in the divergent priming condition, a finding that is consistent with previous literature supporting the inverse relation between activation and creativity. We were expecting to see higher activation after cathodal stimulation, which inhibits DLPFC, and a lower activation after anodal stimulation. Our hypothesis was not met. Individuals had constantly lower skin temperature’s values after cathodal stimulation. Our data seem to be coherent with earlier research on creative performance and arousal (e.g., Martindale, 1977) reporting that, when a task is not creative or creative thinking is not prompted (a case similar to cathodal stimulation, that should inhibit some mechanisms linked to creativity), people tend to show lower levels of arousal. Moreover stressors linked with noise that increase arousal and skin temperature have been reported to be negatively associated with performance in creativity tests (Martindale and Greenough, 1973).

Someone might wonder why priming and brain stimulation did not work together, but lead to opposite results. A possible explanation refers to the fact that literature would lead us to expect lower activation whenever a better creative performance is expected, but this is true only if no additional effort is requested. Brain stimulation may have affected body temperature in relation to creative performance *per se*, whereas the convergent priming contrasted such an influence because it increased the effort needed to perform the creative task since it prompted an attitude which rendered the task harder (so producing an effect similar to what stressors do). Our data suggest that priming and brain stimulation acted independently during the creative task. More research has to be carried out to clarify this finding. It would be interesting and useful to collect more evidence in order to support possible biofeedback application aimed to enhance creative thinking.

Our preliminary study provides new and interesting findings that can contribute to understand creativity from a neuropsychological perspective. Yet, it has several limitations. Further research should replicate the findings with a larger sample, balanced by gender. The small sample size issue could potentially affect the generalizability of the findings. To partially address this problem we used a bootstrapping technique, which uses resampling methods to generate empirical (rather than theoretical) estimates of population distributions. Literature has shown that such methods can be useful in situations where the sample is believed to be a reasonable estimate of the population, but it has a small N. The discussion about a suitable sample size to perform bootstrapping is still active: we decided to follow the suggestions given by Chernick (2007) and Hall (1992) who imply that with a sample size similar to ours it is possible to perform bootstrapping. Even if Chernick argues that this is not always the case (e.g., Chernick, 2007, p. 174), the same author in a recent discussion^2^ supports the logic behind performing a bootstrapping a relatively smaller size.

A different location of the reference electrode should also be tested in any further replication of the study: our choice allowed us to constrain tDCS application to one hemisphere, according to the procedure employed in previous studies. Yet, F3/F4 and the mastoid muscle are pretty close together on the same hemisphere and this proximity may have affected the results.

Stimulating DLPFC allowed us to collect some new data on a specific region of the PFC that is related to creativity and whose role has been less explored. The trend observed by Zmigrod et al. (in press) has been supported by further evidence and additional findings concerning the role of individual differences and physiological activation have been acquired. Future studies, relying on a larger sample, might compare the effects on creativity by stimulating DLPFC and other PFC regions.

## Conflict of Interest Statement

The authors declare that the research was conducted in the absence of any commercial or financial relationships that could be construed as a potential conflict of interest.

## Footnotes

^1^We used bootstrapping to generate empirical (rather than theoretical) estimates of our population distributions. This method can be useful in situations where the sample is believed to be a reasonable estimate of the population, but it has a small N.

^2^http://stats.stackexchange.com/questions/33300/determining-sample-size-necessary-for-bootstrap-method-proposed-method
